# Linking Hydrologic Alteration to Biological Impairment in Urbanizing Streams of the Puget Lowland, Washington, USA^1^

**DOI:** 10.1111/j.1752-1688.2009.00306.x

**Published:** 2009-04

**Authors:** Curtis L DeGasperi, Hans B Berge, Kelly R Whiting, Jeff J Burkey, Jan L Cassin, Robert R Fuerstenberg

**Keywords:** benthic macroinvertebrates, environmental indicators, hydrologic metrics, index of biological integrity, land use/land cover change, urbanization, urban streams, watershed management

## Abstract

We used a retrospective approach to identify hydrologic metrics with the greatest potential for ecological relevance for use as resource management tools (i.e., hydrologic indicators) in rapidly urbanizing basins of the Puget Lowland. We proposed four criteria for identifying useful hydrologic indicators: (1) sensitive to urbanization consistent with expected hydrologic response, (2) demonstrate statistically significant trends in urbanizing basins (and not in undeveloped basins), (3) be correlated with measures of biological response to urbanization, and (4) be relatively insensitive to potentially confounding variables like basin area. Data utilized in the analysis included gauged flow and benthic macroinvertebrate data collected at 16 locations in 11 King County stream basins. Fifteen hydrologic metrics were calculated from daily average flow data and the Pacific Northwest Benthic Index of Biological Integrity (B-IBI) was used to represent the gradient of response of stream macroinvertebrates to urbanization. Urbanization was represented by percent Total Impervious Area (%TIA) and percent urban land cover (%Urban). We found eight hydrologic metrics that were significantly correlated with B-IBI scores (Low Pulse Count and Duration; High Pulse Count, Duration, and Range; Flow Reversals, *T*_Qmean_, and R-B Index). Although there appeared to be a great deal of redundancy among these metrics with respect to their response to urbanization, only two of the metrics tested – High Pulse Count and High Pulse Range – best met all four criteria we established for selecting hydrologic indicators. The increase in these high pulse metrics with respect to urbanization is the result of an increase in winter high pulses and the occurrence of high pulse events during summer (increasing the frequency and range of high pulses), when practically none would have occurred prior to development. We performed an initial evaluation of the usefulness of our hydrologic indicators by calculating and comparing hydrologic metrics derived from continuous hydrologic simulations of selected basin management alternatives for Miller Creek, one of the most highly urbanized basins used in our study. We found that the preferred basin management alternative appeared to be effective in restoring some flow metrics close to simulated fully forested conditions (e.g., *T*_Qmean_), but less effective in restoring other metrics such as High Pulse Count and Range. If future research continues to support our hypothesis that the flow regime, particularly High Pulse Count and Range, is an important control of biotic integrity in Puget Lowland streams, it would have significant implications for stormwater management.

## Introduction

**FIGURE 1:**
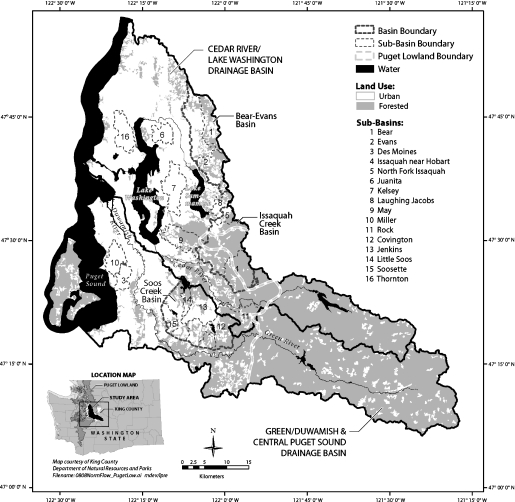
Map of Study Area Showing the 16 Sub-Basins and Urban *vs.* Forested Land Cover.

In highly urbanized lowland areas surrounding Puget Sound, Washington (Figure 1), forest cover has been reduced to a few patches along highly altered stream courses that were not incorporated into the subterranean stormwater conveyance system. In the suburban and rural fringe between these metropolitan areas and the national and state forest lands concentrated along the western flanks of the Cascade Mountains, private forest lands previously managed for timber are being converted to other uses as a consequence of urban sprawl (McClinton and Lassiter, 2002; Alig *et al.*, 2003; Northwest Environmental Forum, 2006; Plantinga *et al.*, 2007). Significant losses of forest are projected to continue with losses concentrated along the fringes of growing metropolitan areas and major transportation corridors (Alig *et al.*, 2003, 2004; Plantinga *et al.*, 2007).

The general phenomenon of conversion of fringe rural resource lands to other uses (primarily suburban development and transportation) is mirrored in metropolitan areas across the globe as the human population grows and is concentrated in cities and expands into the suburban fringes (Alig *et al.*, 2004). A complex interaction of socioeconomic factors drives urban sprawl that includes extension and expansion of urban services to rural areas (e.g., sewer, water, and roads), low timber/agricultural resource values relative to conversion to suburban uses, and lack of consideration of the value of the ecosystem services that healthy forests provide (Alberti *et al.*, 2003; Alig *et al.*, 2004; Alberti, 2005).

The loss of forest cover and development in the Puget Lowland increases winter peak flows and decreases winter base flows as interception and infiltration of rainfall is reduced and runoff from compacted soils and impervious cover is more quickly routed to receiving streams via engineered conveyance networks (Booth, 1991; Booth and Jackson, 1997; Konrad and Booth, 2005). In the absence of mitigation measures, increased runoff typically results in increased frequency and magnitude of flooding and channel erosion (Booth and Jackson, 1997; Konrad *et al.*, 2005). Runoff from impervious surfaces also typically delivers greater amounts of nutrients, sediments, fecal indicator bacteria, and chemical contaminants (Hatt *et al.*, 2004; Carle *et al.*, 2005). In addition to these hydrologic and water quality effects, forest clearing and development result in direct and mostly irreversible loss of habitat, displacement/extirpation of native species, and ecosystem fragmentation and degradation (Vitousek *et al.*, 1997; McKinney, 2002). In the Puget Sound region, the most visible loss of aquatic species partially attributable to urban development has been the reduction and extinction of native salmon populations, and changes in the species assemblages of urbanized streams (Nehlsen *et al.*, 1991; Frissel, 1993; Doberstein *et al.*, 2000; Matzen and Berge, 2008).

A variety of research efforts conducted in the Puget Lowlands of Washington have demonstrated a statistical relationship between human development of the landscape – most typically represented by percent Total Impervious Area (%TIA) – on benthic invertebrate community structure – typically represented by the Pacific Northwest Benthic Index of Biological Integrity (B-IBI) (May *et al.*, 1997; Morley and Karr, 2002; Booth *et al.*, 2004; Alberti *et al.*, 2007). However, the specific environmental changes causing decreasing B-IBI scores with urbanization have not been conclusively identified. Potential changes include water quality impairment, habitat degradation, and hydrologic alteration and more specifically include changes in channel morphology, streambed material, nutrients, migration barriers, water temperature, and water chemistry (Konrad and Booth, 2005). Unfortunately, these variables are generally correlated with each other: multiple, scale-dependent mechanisms are at play; responses to stressors are typically nonlinear; and there are difficulties associated with separating present-day from past effects (Allan, 2004).

Nonetheless, a few authors have suggested that hydrologic alteration is the primary cause of declining biological richness and B-IBI scores as basins become urbanized (Wang *et al.*, 2000; Morse *et al.*, 2003; Booth *et al.*, 2004; Walsh, 2004; Konrad and Booth, 2005; Walsh *et al.*, 2005). The lack of strong correlations between conventional water quality and B-IBI except in the most highly urbanized streams (May *et al.*, 1997) lends further evidence (albeit circumstantial) to the predominant role hydrologic change plays in declining biological health (Booth, 2005).

**FIGURE 2:**
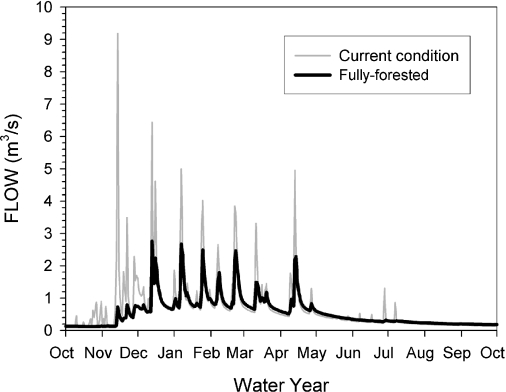
Typical Annual Runoff Pattern (October to September) Under Fully Forested and Highly Urbanized Conditions Derived From Calibrated Kelsey Creek Hydrologic Simulation Program-FORTRAN (HSPF) Model.

Parallel to the increasing suspicion that hydrologic change is a major driver of biological degradation in streams is the recognition that native stream biota are best adapted to the *natural flow regime*– the flow regime typical of the millennia prior to significant human alteration of the landscape (Richter *et al.*, 1996, 1997; Poff *et al.*, 1997). Although the historical flow regime was not without its inter- and intra-annual disturbances, forest clearing and urbanization in the Puget Lowlands over the last 150 years have dramatically altered the historical flow regime, exacerbating disturbances during winter high flows and introducing disturbances during late summer when none typically occurred in the past (Booth, 1991; Konrad *et al.*, 2005– see Figure 2).

A host of hydrologic metrics have been developed to provide quantitative measures of hydrologic change between predisturbance and postdisturbance conditions (e.g., Richter *et al.*, 1997; Clausen and Biggs, 2000). The difficulty lies in identifying hydrologic metrics that respond to urbanization and can also be shown to be biologically relevant (Poff *et al.*, 1997; Konrad, 2001; Bunn and Arthington, 2002; Arthington *et al.*, 2006). Ideally, finding acceptable values for these hydrologic metrics would become the focus for management, rather than a one-size-fits-all approach (Arthington *et al.*, 2006) or a simple (and generally infeasible) requirement to completely restore the predisturbance flow regime.

Our objective was to identify hydrologic metrics for small streams in the Puget Lowlands that would be useful as flow management tools with the greatest potential ecological relevance (i.e., hydrologic indicators). We considered potentially useful stream hydrologic indicators to: (1) be sensitive to land cover change (i.e., forest clearing and subsequent urbanization) consistent with expected hydrologic response, (2) show statistically significant trends over time in response to urbanization, (3) be correlated with measures of biological response to urbanization, and (4) be relatively insensitive (uncorrelated) to potentially confounding variables (e.g., basin area).

We also wished to compare the relative usefulness of various urbanization measures used in previous studies, specifically %TIA, in comparison with the hydrologic metrics that best correlated with B-IBI. We hypothesized that the selected hydrologic indicators would explain much more of the variance in B-IBI scores if these metrics were indeed more direct measures of the influence of urbanization on stream biotic integrity. Our approach was retrospective and relied on available land cover, basin area, continuous flow, and benthic invertebrate data.

Once we had identified a set of hydrologic metrics that we believed had the greatest potential as biologically relevant flow indicators, we performed an initial evaluation of their application by comparing hydrologic indicators derived from continuous hydrologic simulations of selected basin management alternatives for Miller Creek, one of the most highly urbanized basins used in our study. Through an interlocal agreement, a multi-jurisdictional effort was initiated to develop a basin plan that provided recommendations to improve the condition of Miller Creek (Executive Committee, 2006). The goal of the basin plan was to identify measures that would protect the creek from the impacts of existing and future development, specifically aquatic habitat degradation, water quality, and flooding.

Because the Miller Creek basin was so highly urbanized, the basin planning goal was set at restoring flow duration and magnitude to levels reflecting 75% forest, 15% grass, and 10% impervious cover throughout the basin (75/15/10), including preexisting development. The preferred basin management alternative included a requirement that all new development in the basin comply with flow control requirements that achieved runoff rates that matched 75/15/10 conditions and construction of enhanced storage and outlet control at an existing regional detention facility.

## Study Area

The Puget Lowland occupies a glaciated trough trending north-south characterized primarily by permeable outwash deposits in valleys and along stream corridors and less permeable till-capped plateaus. To the west and east, uplands rise to the Olympic and Cascade Mountains which are dominated by bedrock and an overall surficial geology distinct from the lowlands (Booth *et al.*, 2003).

The lowland region experiences a warm and wet maritime climate, although winters are wetter than summers – approximately three-fourths of the annual precipitation falls in October through March. Precipitation ranges from about 1,000 mm per year near sea-level and increases with elevation toward the mountain crests as does the seasonal amount of snowfall. Rainfall (and occasionally rain-on-snow) is the dominant source of streamflow in the Puget Lowland, with highest flows in November through March and lowest flows in July through September (Beechie *et al.*, 2006). During the winter wet season, rainfall is of light to moderate intensity over an extended period – typically one to several days. Puget Lowland 24-hour precipitation with a two-year return frequency is approximately 50 mm with intensity increasing with elevation.

Historically, the Puget Lowland was primarily covered by humid, temperate coniferous forests, which were mostly cleared for timber in the 19th and 20th centuries. Suitable lowlands and plateaus were converted to farmland. Denser settlements concentrated in lowlands near lakes, rivers, and streams. Approximately four million people currently live in the Puget Sound basin and over a million more inhabitants are expected in the next decade, primarily in urban and suburban areas of the Puget Lowland. Currently, second growth forests (often mixed with deciduous trees) are rapidly being converted to residential and commercial uses with associated losses of canopy interception and soil moisture storage and increases in impervious cover.

Historically, conveyance systems in developing areas were designed to route rainfall runoff more rapidly to streams, although progressively more protective regulations have resulted in increasing amounts of mitigation – primarily on-site and regional flow detention facilities aimed initially at the control of peak flows and currently aimed at matching predevelopment flow-duration curves (Booth and Jackson, 1997; Booth *et al.*, 2002). The most recent regulations in King County, which only apply to unincorporated rural areas, emphasize retention of forest cover, minimization of impervious surface cover, and minimization of soil compaction/removal (http://www.kingcounty.gov/property/permits/codes/CAO.aspx). Nonetheless, most development and retrofit attempts in the region predate the most recent regulations.

## Methods

### Site Selection

We used a “space-for-time” approach that assumes that spatial variation in the degree of urbanization of subcatchments captures the historical temporal trend in urbanization in the study area (Keenan and Ayers, 2002; Morse *et al.*, 2003; Roy *et al.*, 2003; Booth *et al.*, 2004; Fitzpatrick *et al.*, 2004– but see Wang *et al.*, 2000). Major assumptions of this approach include that the spatial character of development reflects the process of development over time and that other confounding factors such as underlying geology, climate, or topography are controlled as much as possible by confining the study area to a relatively homogeneous physical and biological environment.

As the hypothesis is that the hydrologic regime is of overarching importance in controlling the character of biological communities in Puget Lowland streams, we broadened our selection of sites beyond those that have typically been considered in previous studies (Morley and Karr, 2002; Booth *et al.*, 2004; Cassin *et al.*, 2005; Alberti *et al.*, 2007) to include a relatively undeveloped higher elevation sub-basin in the Issaquah Creek basin near Hobart, Washington, with significant amounts of bedrock that might mimic the effects of impervious cover on streamflow (i.e., increase the frequency and magnitude of winter storm flow and generate peak flows during summer). Although the stream monitoring site in the Issaquah Creek basin is within the Puget Lowland boundaries, the upper drainage of the sub-basin includes higher elevation areas that result from the farthest westward intrusion of the Cascade mountain range into the Puget Lowland (Booth *et al.*, 2003).

The primary limitation on the number of paired biology-hydrology sites used in our study was the availability of continuous gauge records. A total of 16 locations were identified in 11 major creek basins in King County that had at least one complete water year (October to September) and calendar year of continuous flow data coincident with the benthic macroinvertebrate sampling year (Figure 1, Table 1). It should be noted that these streams have not historically received any direct discharges of treated wastewater from regional treatment facilities and are assumed to be affected primarily by the conversion of upland forests to residential and commercial uses.

**TABLE 1:** Continuous Flow Monitoring Time Period and Benthic Index of Biological Integrity (B-IBI) Sampling Year.

		Stream Gauging		B-IBI	
Map ID	Location	Gauge ID	Available Data	Sample ID	Year
1	Bear	02e^1^	1995-2006	BB975^3^	1997
2	Evans	18a^1^	1988-2007	BBEVN1^1^	1999
3	Des Moines	11d^1^	1992-2007	DM_1995^4^	1995
4	Issaquah near Hobart	12120600^2^	1987-2007	ISISS4^1^	1995
5	North Fork Issaquah	46a^1^	1989-2007	ISNF1^1^	1996
6	Juanita	27a^1^	1993-2006	JU_1995^4^	1995
		12120500^2^	1964-1989		
7	Kelsey	12120000^2^	1956-2007	KE_1995^4^	1995
8	Laughing Jacobs	15c^1^	1992-2007	LJ98us^3^	1998
9	May	37a^1^	1990-2007	MA971^3^	1997
10	Miller	42a^1^	1992-2007	MI971^3^	1997
11	Rock	31l^1^	1996-2006	RO982^3^	1998
12	Covington	09a^1^	1989-2007	SOOS04^1^	1995
13	Jenkins	26a^1^	1989-2007	JE971^3^	1997
14	Little Soos	54i^1^	1996-2007	SOOS08^1^	1997
15	Soosette	54h^1^	1995-2007	SOOS06a^1^	1995
16	Thornton	12128000^2^	1997-2007	TH98DS^3^	1998

Notes: Map ID refers to basins identified in Figure 1.

1 King County.

2 USGS.

3 Morley (2000).

4 J. Karr, data obtained from SalmonWeb http://www.cbr.washington.edu/salmonweb/, *accessed* January 18, 2006..

### Benthic Index of Biological Integrity

We selected the 10-metric Pacific Northwest B-IBI recommended by Karr (1998) as our measure of stream biological condition due to its historical use in assessments of urbanization impacts on Puget Lowland streams. The Pacific Northwest B-IBI represents four broad community characteristics that include taxa richness and composition (five metrics – total taxa richness, Ephemeroptera taxa richness, Plecoptera taxa richness, Trichoptera taxa richness, number of long-lived taxa), tolerant and intolerant taxa (two metrics – number of intolerant taxa, percent tolerant individuals, excluding chironomids), functional groups (two metrics – number of clinger taxa, percent predator individuals), and percent dominance of the three most abundant taxa (one metric) (Fore *et al.*, 1996; Morley and Karr, 2002).

At each site, three replicate samples were collected within a single riffle using a Surber sampler (Morley and Karr, 2002). Samples were collected in late summer when rainfall is less frequent and intense, antecedent soil moisture is lowest, and flows are expected to be relatively stable. Taxa richness is also high at this time of year and sites are easy to access (Fore *et al.*, 1996). Taxonomic and classification results for the three replicates were averaged and then assigned a value of 1, 3, or 5 for each metric value. The ten metric values were then summed resulting in a total B-IBI score that ranges from 10 (considered very poor biological condition) to 50 (considered excellent biological condition).

Due to the retrospective nature of our study, B-IBI data (Table 1) were not always collected in the immediate vicinity of the stream gauging location. The farthest B-IBI sampling location occurred in the Kelsey Creek basin and was almost 2 km upstream of the gauging site with almost 55% of the basin drainage occurring between the B-IBI site and the gauge. However, the level of development above the B-IBI sampling location and between the B-IBI station and the gauge was very similar – in terms of %TIA the difference was less than 1%. The remaining B-IBI sampling locations were typically much closer, within 1 km of their respective gauging locations and ranged from less than 1-14% of the total drainage area of the basin.

### Basin Characteristics

A variety of geographic information system datasets were available to characterize the level of urbanization, surficial geology, and other physical characteristics of the catchment area upstream of the stream gauging sites. Because previous assessments of urbanization impacts on Puget Lowland streams (Morley and Karr, 2002; Booth *et al.*, 2004; Alberti *et al.*, 2007; Matzen and Berge, 2008) used a 1998 Landsat image classified into seven land cover categories (three urban, bare earth, forested, grass/shrub, and open water) (Hill *et al.*, 2003; Alberti *et al.*, 2007) and the selected benthic invertebrate sampling years ranged from 1995 to 1999, we used the same land cover dataset to calculate percent TIA, urban, and non-urban forest land cover. Percent urban (%Urban) land cover was calculated as the proportion of land cover in each sub-basin occupied by the three urban categories (forested urban, grass/shrub urban, and paved urban) represented in the classified image. Percent Forest (%Forest) was calculated as the proportion of forested land in non-urban areas. Percent TIA (%TIA) was based on estimates in Hill *et al.* (2003) of impervious area within each of the seven land cover classes.

We also used available electronic maps of surficial geology (Booth *et al.*, 2002), elevation (high resolution LiDAR) (King Co. and Puget Sound LiDAR Consortium and USGS National Elevation Database), impervious surface cover based on remote multispectral imaging conducted in 2000 (King County, 2004), and mean annual precipitation (Daly *et al.*, 2002) to further characterize the study basins. Basin characteristics derived from these sources included percent cover of till, outwash, and bedrock exclusive of impervious surface cover (by using the 2000 impervious surface cover data as a mask), mean basin slope and elevation, local channel slope, and mean annual precipitation.

### Hydrologic Metrics

We obtained daily streamflow data from the King County Hydrologic Information Center (http://green.kingcounty.gov/wlr/waterres/hydrology/) and USGS National Water Information System stream gauging databases (http://www.waterdata.usgs.gov/nwis) (Table 1).

We selected a limited number of hydrologic metrics for use in our analysis based on an initial evaluation of a much larger suite of metrics by Cassin *et al.* (2005). We reduced the list further by stipulating that the selected metrics could be calculated with a single year of daily mean flow data – water year or calendar year depending on the metric. The final list consisted of 15 metrics that included representatives from the major flow regime categories of magnitude, duration, timing, frequency, rate of change, and flashiness/variability. A list of the hydrologic metrics evaluated and a description of how they are calculated and their expected response to urbanization is provided in Table 2.

**TABLE 2:** Description of the 15 Hydrologic Metrics Used in This Study.

Component	Metric Name	Definition	Expected Response to Urbanization	Units	Reference
	**Low Flow Pulse**	Occurrence of daily average flows that are equal to or less than a threshold set at 50% of the long-term daily average flow rate	Summer low flows are more frequently interrupted by higher storm flows		

Frequency	Low pulse count	Number of times each calendar year that discrete low flow pulses occurred	Increase	Count	Richter *et al.* (1996, 1997, 1998)
Duration	Low Pulse Duration	Annual average duration of low flow pulses during a calendar year	Decrease	Days	Richter *et al.* (1996, 1997, 1998)
Duration	Low Pulse Range	Range in days between the start of the first low flow pulse and the end of the last low flow pulse during a calendar year	Decrease	Days	This study

	**High Flow Pulse**	Occurrence of daily average flows that are equal to or greater than a threshold set at twice (two times) the long-term daily average flow rate	High flow pulses occur more frequently and although flow magnitudes are higher, high pulse durations are shorter		
Frequency	High Pulse Count	Number of days each water year that discrete high flow pulses occur	Increase	Count	Richter *et al.* (1996, 1997, 1998)
Duration	High Pulse Duration	Annual average duration of high flow pulses during a water year	Decrease	Days	Richter *et al.* (1996, 1997, 1998)
Duration	High Pulse Range	Range in days between the start of the first high flow pulse and the end of the last high flow pulse during a water year	Increase	Days	This study

	**Various**				

Rate of Change	Fall Rate	The average rate of fall of all falling portions of the daily hydrograph during a calendar year	Increase	m^3^/s per day	Richter *et al.* (1996, 1997, 1998)
Rate of Change	Rise Rate	The average rate of rise of all rising portions of the daily hydrograph during a calendar year	Increase	m^3^/s per day	Richter *et al.* (1996, 1997, 1998)
Frequency	Fall Count	The number of days of declining daily average flows during a calendar year. A decline in flow is counted only when the rate of change is greater than −10%	Increase	Count	Richter *et al.* (1996, 1997)
Frequency	Rise Count	The number of days of increasing daily average flows during a calendar year. An increase in flow is counted only when the rate of change is greater than 10%	Increase	Count	Richter *et al.* (1996, 1997)
Frequency	Flow Reversals	The number of times that the flow rate changed from an increase to a decrease or vise versa during a water year. Flow changes of less than 2% are not considered	Increase	Count	Richter *et al.* (1998)
Flashiness	*T*_Qmean_	The fraction of time during a water year that the daily average flow rate is greater than the annual average flow rate of that year	Decrease	Fraction of year	Konrad and Booth (2002), Konrad (2000)
Flashiness	R-B Index	Richards-Baker Flashiness Index – A dimensionless index of flow oscillations relative to total flow based on daily average discharge measured during a water year	Increase	Unitless	Baker *et al.* (2004)
Magnitude	Seven-day minimum	Centered seven-day moving average annual (calendar year) minimum flow	Decrease	m^3^/s	Richter *et al.* (1996, 1997, 1998)
Timing	Date of annual minimum	Julian day of the date of the minimum daily average flow during a calendar year	Earlier	Julian date	Clausen and Biggs (2000); Richter *et al.* (1996, 1997, 1998)

Note: All metrics based on daily average flow.

Eleven of our metrics were derived from metrics used in the Indicators of Hydrologic Alteration (IHA) (Richter *et al.*, 1996, 1997, 1998). We developed two additional metrics from the IHA high and low pulse metrics –*low pulse range* and *high pulse range*, which measure the span of time in days between the occurrence of the first and last pulse in each calendar year (low pulses) and water year (high pulses).

Two metrics (*T*_Qmean_ and Richards-Baker Flashiness Index or R-B Index) came from previous studies that focused on evaluating regional patterns and trends in flow flashiness related to changes in land cover/land use (Konrad and Booth, 2002; Baker *et al.*, 2004). *T*_Qmean_ has been used to detect trends in flow flashiness related to basin urbanization in the Puget Lowland (Konrad and Booth, 2002).

The IHA low and high pulse metrics typically require an estimate of predisturbance (prior to forest clearing and urbanization in the context of this study) mean flow to determine the exceedance thresholds for identifying high and low flow pulses. Because none of our gauge records predate the period of initial forest clearing or urbanization and urbanization has proceeded continuously in these basins since gauging began (Konrad and Booth, 2002), we relied on available gauging data to estimate the mean flow and pulse thresholds. We use 2 and 0.5 times the gauged mean flow as thresholds for high (above threshold) and low (below threshold) pulses, respectively. We believe using gauged mean flow as the basis of the pulse thresholds is reasonable given the uncertainty in predisturbance mean flow and evidence that the mean flow is not significantly altered by urban development in Puget Lowland streams (Konrad and Booth, 2002).

An inherent assumption in our approach is that the biological responses to changes in hydrology occur over multi-year time scales (Konrad *et al.*, 2005). Due to the lack of sufficient hydrologic data to thoroughly evaluate the appropriate temporal averaging period for hydrologic metrics in relation to B-IBI scores, we chose to average our hydrologic metrics over three-years (preceding, but including the calendar year in which the B-IBI sample was collected) when possible based on the consideration that the long-lived taxa (typically Plecoptera or stonefly genera) may live more than three years in a stream (Stewart and Stark, 1988). However, some gauging data at some sites were only available for the year the B-IBI sample was collected. Study basins, referenced to Figure 1, data source, and years of available flow data are provided in Table 1.

### Data Analysis

We evaluated the relationship among the various hydrologic metrics, land cover, and B-IBI scores of the study streams by constructing bivariate correlation tables (Pearson’s *r*). We chose to use a parametric approach, rather than a nonparametric approach, because our ultimate goal is the development of predictive models, which rank correlation can not provide. Data that did not meet the assumptions for parametric analysis were normalized using either log_10_ (Low and High Pulse Count, Low and High Pulse Duration, and Fall and Rise Rate) or arcsine square root (%TIA, %Urban, and %Forest) transformations. We only discuss variables that were statistically significant based on the Benjamini and Hochberg False Discovery Rate control procedure (Verhoeven *et al.*, 2005) to control the probability of identifying spurious correlations (Type I error), while minimizing the number of Type II errors.

Sensitivity of hydrologic metrics with a significant correlation with B-IBI to potentially confounding variables was assessed by evaluating the correlations between each hydrologic metric and various basin characteristics – Basin Area, %Outwash, %Till, Basin Elevation, Local Channel Slope, and Precipitation. Data that did not meet the assumptions for parametric analysis were normalized using either log_10_ (Local Channel Slope) or arcsine square root (%Outwash and %Till) transformations. Transformations for %Bedrock and Basin Slope did not result in a normal distribution and were not evaluated. Interactions between measures of urbanization and B-IBI and basin characteristics were also evaluated in the correlation matrix.

Principal components analysis (PCA) was conducted on the correlation matrix of the hydrologic metrics with a significant correlation with B-IBI to evaluate the major modes of variation and potential redundancy (Clausen and Biggs, 2000; Olden and Poff, 2003). We did not include the complete list of hydrologic metrics in the PCA because we wanted to focus only on those metrics that showed a strong association with a measure of biological response to urbanization. We used simple linear regression to illustrate the strength of relationships between the dependent variable B-IBI or land cover metrics (%TIA, %Urban, and %Forest) and independent hydrologic metrics, including the first principal component (PC) of the hydrologic metric PCA (Clausen and Biggs, 1997).

We also evaluated the utility of the hydrologic metrics that showed the strongest correlations with B-IBI scores to detect the hydrologic impacts of urbanization. We performed nonparametric Mann-Kendall trend tests on long-term datasets from two of our study basins that have undergone rapid urbanization over the periods of their hydrologic records (Juanita and Kelsey) and one basin that is still relatively undeveloped (Issaquah). We assumed that significant trends in developing basins provide evidence for a cause-and-effect relationship between urbanization and hydrologic alteration and should corroborate the space-for-time correlations with measures of urbanization. Establishing connections between urbanization and these metrics and evaluating trend detection capabilities provides support for the usefulness of these metrics from a management perspective for long-term trend detection monitoring.

### Miller Creek Basin Plan Case Study

Continuous hydrologic model results were produced using a 50-year hourly rainfall record as part of the planning process to simulate hydrology under four conditions: (1) fully forested, (2) basin-wide 75/15/10 development (the planning goal), (3) current conditions (based on 1995 land cover), and (4) the preferred basin management alternative described above – application of 75/15/10 flow control requirements for all new development and enhanced detention capacity. To evaluate the utility of our selected hydrologic indicators in basin planning, using the Miller Creek Basin Plan as a test case, we summarized and compared the long-term average (50 years) of selected metrics under the four modeled conditions.

Traditionally, these basin flow management models (developed using Hydrologic Simulation Program-FORTRAN; HSPF) have been calibrated to predict the timing, magnitude, and duration of winter peak flow events (and annual and seasonal flow volumes) as a result of historical focus on the control of flooding and channel erosion. The ability of these models to predict various hydrologic metrics has not been systematically tested. Therefore, we used a nonparametric Mann-Whitney *U*-test to evaluate the null hypothesis that the difference in central-tendency of the model-predicted and observed annual hydrologic metrics is zero. A probability level of <0.05 was used to test the null hypothesis and conclude that the Miller Creek Basin Plan model could reproduce the central-tendency of a particular flow metric. Comparison of modeled hydrologic indicators among the four modeled conditions was limited to those metrics that could be reliably predicted by the model.

## Results

**FIGURE 3:**
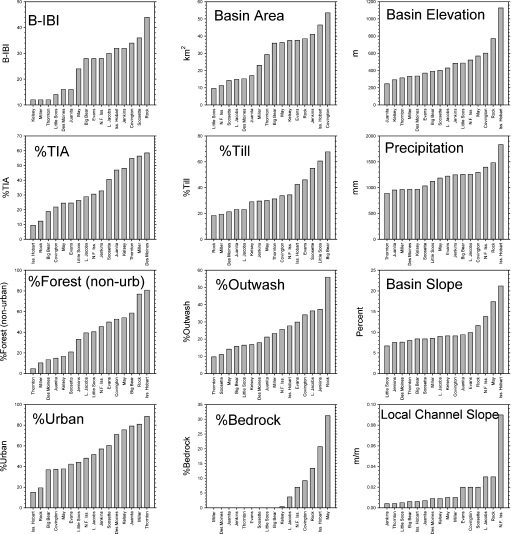
Bar Charts Illustrating Distribution of Sub-Basin Characteristics for the 16 Sub-Basins Used in This Study.

Stream biological condition as measured by the B-IBI ranged from 12 (*very poor*) to 44 (*good*) out of a possible range of 10 to 50 (Figure 3). No sites were classified as being in *excellent* condition (B-IBI ≥ 46). Only one site (Rock Creek; B-IBI = 44) was in *good* condition (B-IBI ≥ 36) and five sites were classified as in *very poor* condition (B-IBI ≤ 16).

The basin areas represented by the selected gauging locations ranged from 10 to 54 km^2^ (Figure 3). The measures of urbanization (%TIA and %Urban) did not include any minimally disturbed basins (i.e., forest dominated basins) but did include a range of urbanization from relatively undeveloped rural to some of the most highly urbanized basins with intact streams (Miller and Des Moines Creeks) (Figure 3). The degree of urbanization represented by %TIA and %Urban, ranged from 10% to 59% and 15% to 89%, respectively. Issaquah Creek near Hobart and Rock Creek were the least urbanized and had the highest (∼80%) non-urban forest cover. The surficial geology of the study basins is dominated by till and outwash deposits, although seven sub-basins that drain the westernmost extension of the Cascades also contained from 4% to 31% bedrock (Figure 3). With regard to %Outwash, Rock Creek stood out among the other basins with 56% of the basin in outwash deposits (Figure 3). Table 3 lists the mean and range of all measured landscape variables across the sites.

**TABLE 3:** Summary Statistics for Basin Characteristics and Hydrologic Metrics Calculated for 16 Stream Basins.

Variable	Description (units)	Minimum	Mean	Maximum
Basin characteristics				
Basin area	Drainage basin area (km^2^)	9.6	28.8	53.5
%TIA	1998 total impervious area (%)	9.5	33.5	58.5
%Outwash	Surficial outwash deposits (%)	9.7	24.6	56
%Till	Surficial till deposits (%)	18.4	35.3	67.7
%Bedrock	Surficial bedrock cover (%)	0	5.4	31.2
%Urban	1998 urban land cover (%)	15.1	52.8	88.7
%Forest (non-urban)	1998 non-urban forest cover (%)	4.8	38.2	80.8
Basin elevation	Mean basin elevation (m)	75	146	344
Precipitation	Mean annual precipitation (mm)	891	1192	1833
Basin slope	Mean basin slope (%)	6.7	10.0	21.2
Local channel slope	Local channel slope (m/m)	0.004	0.018	0.090
Hydrologic metrics				
Q_mean_	Mean annual average flow (m^3^/s)	0.153	0.490	1.267
Low Pulse Count	Number of low pulse events per year (count)	2	10	28
Low Pulse Duration	Mean duration of low pulse events (days)	7	26	93
Low Pulse Range	Range each calendar year over which low pulse events occur (days)	67	207	341
High Pulse Count	Number of high pulse events per year (count)	2	10	22
High Pulse Duration	Mean duration of high pulse events (days)	2	7	31
High Pulse Range	Range each Water Year over which high pulses occur (days)	34	168	306
Fall Rate	Average fall rate of falling flows (m^3^/s per day)	0.035	0.098	0.244
Rise Rate	Average rise rate of rising flows (m^3^/s per day)	0.059	0.190	0.518
Fall Count	Number of falling flows (count)	25	92	137
Rise Count	Number of rising flows (count)	42	68	86
Flow Reversals	Number of flow reversals per year (count)	37	55	70
*T*_Qmean_	Fraction of year that daily flow exceeds mean annual flow (fraction of year)	0.25	0.31	0.38
R-B Index	Richards-Baker Flashiness Index (unitless)	0.08	0.27	0.49
Seven-day minimum	Minimum seven-day moving average flow (m^3^/s)	0.005	0.092	0.283
Date of annual daily minimum	Date of annual daily minimum flow (Julian date)	146	239	311

Note: Hydrologic statistics based on up to three years of data collected in years prior to and including the year benthic invertebrate samples were collected.

Basin mean annual flow ranged from 0.153 to 1.267 m^3^/s, primarily reflecting the variation in basin drainage area (Table 3). The selected hydrologic metrics presented a fairly wide range of values that we hypothesize are primarily the result of the range of levels of urbanization in our study basins (Table 3).

Data for the individual basins, including B-IBI scores, basin characteristics, and mean values for the 15 hydrologic metrics are provided in Table S1.

### Relationships Between Benthic Index of Biological Integrity, Land Cover, and Hydrologic Metrics

We found statistically significant negative correlations between B-IBI and %TIA (*r* = −0.733; *p*<0.01) and B-IBI and %Urban (*r* = −0.748; *p*<0.01) and a significant positive correlation between B-IBI and %Forest (non-urban) (*r* = 0.731; *p*<0.01) (Table 4). Each land cover metric explained roughly half of the variance in B-IBI scores.

**TABLE 4:** Pearson Correlation of Hydrologic Metrics With Measures of Urbanization, Non-Urban Forest Cover, and Benthic Index of Biological Integrity Scores in 16 Stream Basins.

	arcsin sqrt (%Total Impervious Area)		arcsin sqrt (%Urban)		arcsin sqrt (%Forest)		Benthic Index of Biological Integrity	
	*r*	*p*	*r*	*p*	*r*	*p*	*r*	*p*
				
Benthic Index of Biological Integrity	−0.733	0.001	−0.748	0.001	0.731	0.001		
log (Low Pulse Count)	0.530	0.035	0.443	0.086	−0.450	0.080	−**0.664**	**0.005**
log (Low Pulse Duration)	−**0.587**	**0.017**	−**0.589**	**0.016**	0.559	0.025	**0.766**	**0.001**
Low Pulse Range	0.087	0.750	−0.085	0.754	0.009	0.973	−0.036	0.896
log (High Pulse Count)	**0.700**	**0.003**	**0.757**	**0.001**	−**0.716**	**0.002**	−**0.844**	**<0.0001**
log (High Pulse Duration)	−**0.638**	**0.008**	−**0.638**	**0.008**	**0.634**	**0.008**	**0.801**	**0.0002**
High Pulse Range	**0.772**	**<0.001**	**0.807**	**<0.001**	−**0.759**	**0.001**	−**0.854**	**<0.0001**
log (Fall Rate)	0.023	0.932	0.048	0.859	−0.003	0.990	−0.317	0.231
log (Rise Rate)	0.001	0.997	0.020	0.941	0.024	0.931	−0.265	0.321
Fall Count	0.426	0.100	0.372	0.156	−0.402	0.123	−0.420	0.106
Rise Count	0.543	0.030	0.506	0.046	−0.530	0.035	−0.529	0.035
Flow Reversals	**0.688**	**0.003**	**0.681**	**0.004**	−**0.696**	**0.003**	−**0.652**	**0.006**
*T*_Qmean_	−0.527	0.036	−0.455	0.077	0.491	0.053	**0.685**	**0.003**
R-B Index	**0.736**	**0.001**	**0.645**	**0.007**	−**0.683**	**0.004**	−**0.703**	**0.002**
Seven-day minimum flow	−0.266	0.319	−0.188	0.485	0.245	0.360	0.057	0.835
Date of annual daily minimum	−0.364	0.165	−0.296	0.266	0.381	0.145	0.475	0.063

Note: Values given in boldface indicate significance based on Benjamini and Hochberg False Discovery Rate control (*p*=0.05; *k*=15) (Verhoeven *et al.*, 2005).

Six of the 15 hydrologic metrics (Low Pulse Duration, High Pulse Count, High Pulse Duration, High Pulse Range, Flow Reversals, and R-B Index) were significantly correlated (based on the Benjamini and Hochberg False Discovery Rate control procedure) with %TIA and %Urban (Table 4). The strongest correlations were between High Pulse Duration and %TIA (*r* = 0.772; *p*<0.001) and %Urban (*r* = 0.807; *p*<0.001). Weaker, but significant, correlations were found with Low Pulse Duration and %TIA (*r* = −0.587; *p*<0.05) and %Urban (*r* = −0.589; *p*<0.05). Similar correlations with opposite signs were found between all but one (Low Pulse Duration) of the same six hydrologic metrics and %Forest (Table 4). The signs of the statistically significant correlations were consistent with their expected response to urbanization (Table 2).

Eight of the 15 hydrologic metrics (Low Pulse Count, Low Pulse Duration, High Pulse Count, High Pulse Duration, High Pulse Range, Flow Reversals, *T*_Qmean_, and R-B Index) were significantly correlated with B-IBI (Table 4). The strongest correlation was with High Pulse Range (*r* = −0.854; *p* < 0.0001) and the weakest statistically significant correlation was with Flow Reversals (*r* = −0.652; *p* < 0.01). The sign of the significant correlations was consistent with the expected biological response to these metrics – B-IBI scores increased in response to fewer Low Pulse and High Pulse Counts and Flow Reversals, shorter High Pulse Range, longer High Pulse and Low Pulse Duration, higher *T*_Qmean_, and lower R-B Index. Only six of these hydrologic metrics were also significantly correlated with the urbanization metrics. Low Pulse Count and *T*_Qmean_ were significantly correlated with B-IBI but not with the urbanization metrics.

### Potential Confounding Variables

Several basin characteristics (%Outwash, Basin Elevation, and Precipitation) were significantly correlated (*p*<0.05) with the eight potential hydrologic indicators, but these basin characteristics were also significantly correlated with measures of urbanization and B-IBI scores. Local Channel Slope was significantly correlated with High Pulse Duration (*r* = 0.513; *p*<0.05) and Basin Area was significantly correlated with Flow Reversals, *T*_Qmean_, and R-B Index (*r* = −0.523; *p*<0.05; *r* = 0.685; *p*<0.05; *r* = −0.503; *p*<0.05, respectively) but not with urbanization measures or B-IBI scores. Percent Till was not significantly correlated with the eight hydrologic metrics or with measures of urbanization or B-IBI scores. Unfortunately, no transformation resulted in a normal distribution of %Bedrock or Basin Slope, so evaluating the response of the hydrologic metrics to these basin variables was not possible.

### Principal Components Analysis

The PCA showed that the first four PCs explained 94% of the total variance in the selected flow metrics (Table 5). The first and second PCs (PC 1 and PC 2) explained 76.4 and 9.1%, respectively, of the total variance. Only the first PC had an eigenvalue greater than 1.0, indicating that PC1 explains more variance than any single predictor would (Kaiser, 1960). All eight metrics load strongly (|*r*| > 0.8) on PC 1, although High Pulse Count and High Pulse Range also load less strongly on PC 2 (0.423 and 0.418, respectively).

**TABLE 5:** Principal Component Analysis Loadings (PC 1 through 4) of the Eight Hydrologic Metrics That Were Significantly Correlated With Benthic Index of Biological Integrity Scores in 16 Study Basins.

Variable	PC 1	PC 2	PC 3	PC 4
log (Low Pulse Count)	0.883	−0.341	0.070	0.005
log (Low Pulse Duration)	−0.930	−0.172	−0.066	−0.121
log (High Pulse Count)	0.866	0.423	−0.196	−0.115
log (High Pulse Duration)	−0.836	−0.010	−0.397	0.359
High Pulse Range	0.874	0.418	−0.139	0.030
Flow Reversals	0.860	−0.032	0.234	0.389
*T*_Qmean_	−0.827	0.393	0.328	0.067
R-B Index	0.915	−0.269	−0.103	−0.025
Eigenvalue	6.116	0.728	0.398	0.314
Explained variance (%)	76.4	9.1	5.0	3.9
Cumulative explained variance (%)	76.5	85.5	90.5	94.4

**FIGURE 4:**
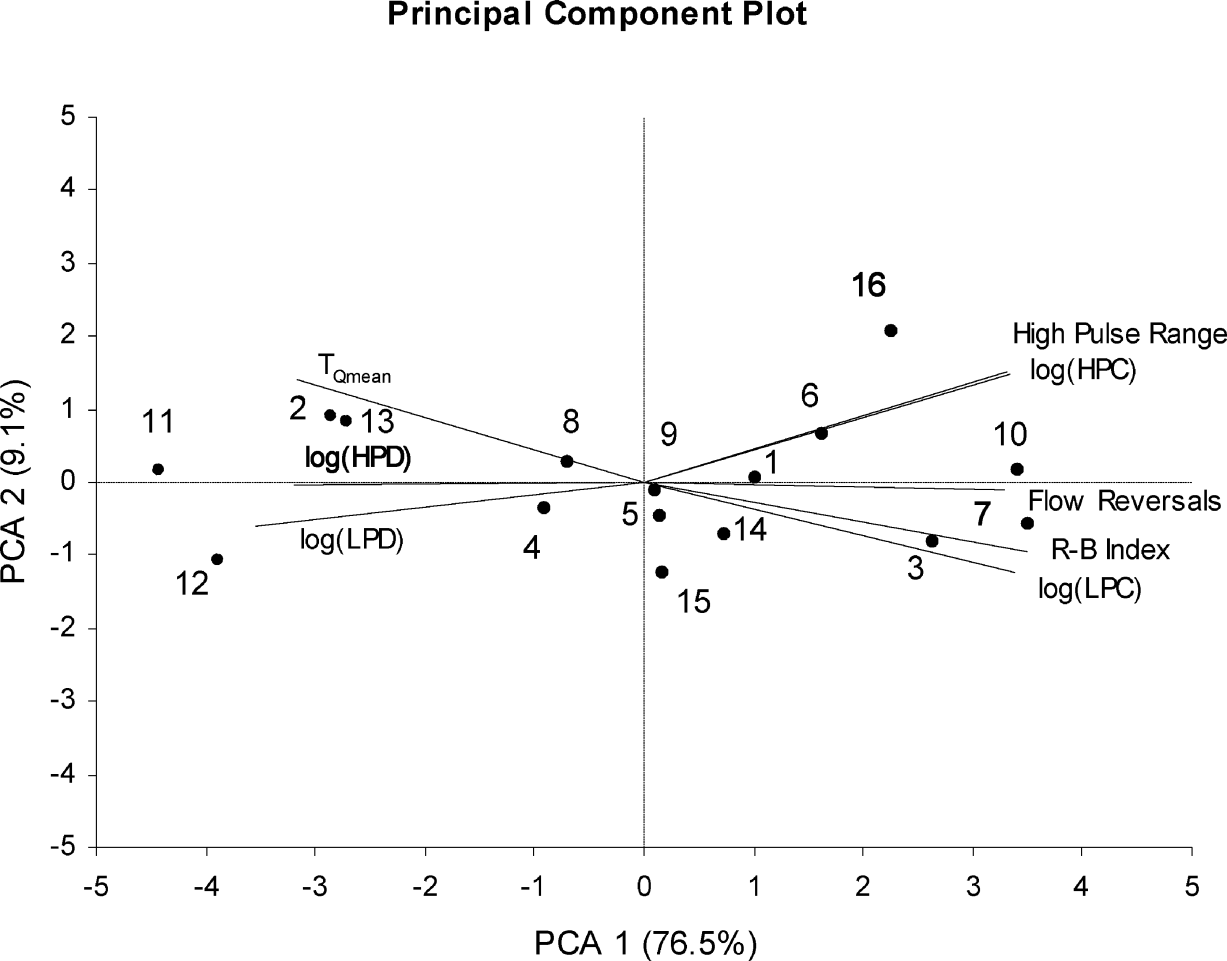
Plot of First Two Principal Components From Principal Component Analysis of the Eight Hydrologic Metrics That Were Significantly Correlated With Benthic Index of Biological Integrity Scores. Numbers next to symbols are the basin map numbers provided in Table 1.

From Figure 4 and Table 5, we infer that PC 1 reflects an urbanization gradient associated with a biological response that is significantly correlated with all of the metrics – increasing urbanization results in more High and Low Pulse Counts, shorter High and Low Pulse Duration, longer High Pulse Range, higher R-B Index, more Flow Reversals, and lower *T*_Qmean_. In general, there is a great deal of redundancy with respect to the response to urbanization among these eight hydrologic metrics. PC 2 reflects a gradient orthogonal to PC 1 that is most strongly related to changes in High Pulse Count and Range.

### Trends in Hydrologic Metrics

**FIGURE 5:**
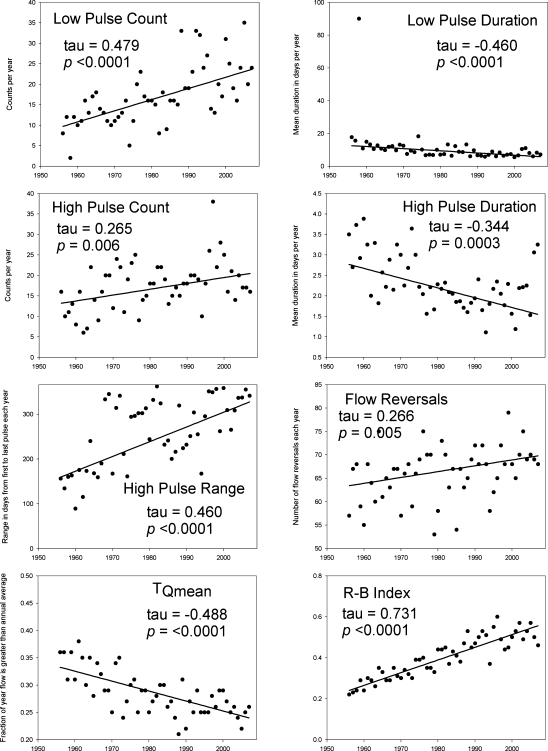
Time Series Plots Showing Kelsey Creek Trends in the Eight Hydrologic Metrics That Were Significantly Correlated With Benthic Index of Biological Integrity Scores. Statistically significant trends in all eight hydrologic metrics were identified in Kelsey Creek, a basin which has one of the longest complete daily hydrologic records (1956-2003) that cover a period of rapid urbanization.

The eight hydrologic metrics that were significantly correlated with B-IBI also demonstrated statistically significant historical trends in at least one of the two urbanized basins (Kelsey and Juanita – see Figure 1) and only one significant trend in the relatively rural basin (Issaquah) selected for long-term trend analysis (Table 6). All eight metrics demonstrated trends consistent with the expected hydrologic response to urbanization in Kelsey Creek (Figure 5), a highly urbanized basin in the county with the longest period of flow observations. Six of the eight hydrologic metrics (Low Pulse Count, Low Pulse Duration, High Pulse Range, High Pulse Count, *T*_Qmean_, and R-B Index) demonstrated statistically significant trends in Juanita Creek (Table 6).

**TABLE 6:** Summary of Mann-Kendall Test for Trend in Selected Hydrologic Metrics in Two Rapidly Urbanizing and One Relatively Undeveloped Basin.

	Kelsey^1^		Juanita^2^		Issaquah^3^	
	tau	*p*	tau	*p*	tau	*p*
Low Pulse Count	**0.479**	**<0.0001**	**0.400**	**0.004**	−0.083	0.43
Low Pulse Duration	−**0.460**	**<0.0001**	−**0.348**	**0.01**	0.156	0.14
High Pulse Count	**0.265**	**0.006**	**0.332**	**0.02**	−0.090	0.39
High Pulse Duration	−**0.344**	**0.0003**	−0.219	0.12	−0.202	0.055
High Pulse Range	**0.460**	**<0.0001**	**0.397**	**0.005**	−0.032	0.77
Flow Reversals	**0.266**	**0.005**	0.234	0.10	−0.170	0.10
*T*_Qmean_	−**0.488**	**<0.0001**	−**0.326**	**0.02**	−0.093	0.38
R-B Index	**0.731**	**<0.0001**	**0.665**	**<0.0001**	**0.208**	**0.046**

Notes: Metrics calculated from long-term gauging records in Kelsey, Juanita, and Issaquah creeks. Values given in boldface indicate significance (*p*<0.05).

1 Kelsey Creek USGS 12120000 (1956-2007).

2 Juanita Creek USGS 12120500 (1964-1989).

3 Issaquah Creek USGS 12121600 (1964-2007).

### Comparison of Predictive Capability of Selected Metrics

**FIGURE 6:**
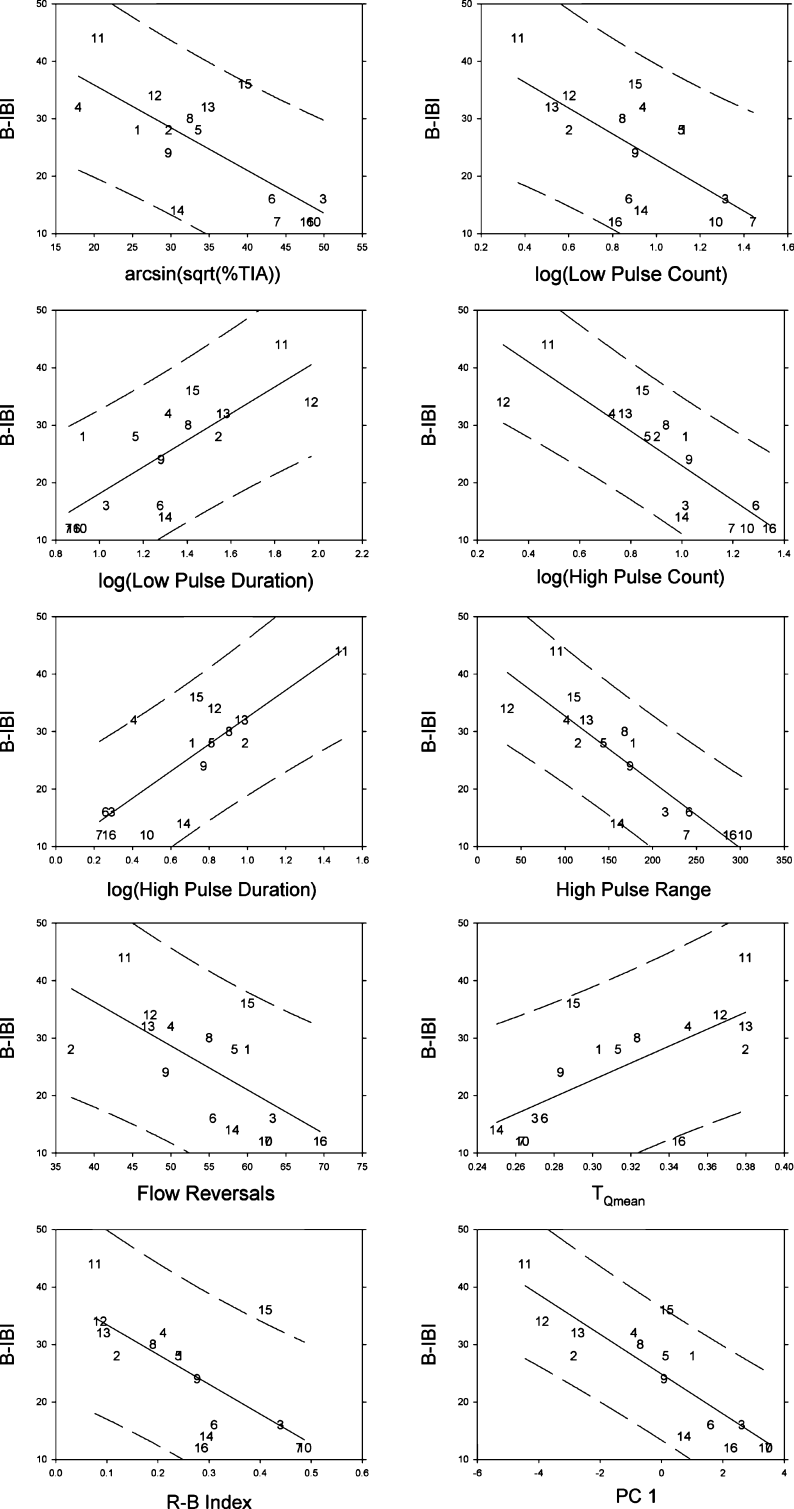
Regression of Selected Hydrologic Metrics and Hydrologic PC1 *vs*. Benthic Index of Biological Integrity Scores. Plots include 95% prediction confidence intervals. Symbols are the basin map numbers provided in Table 1.

Although Table 4 provides a comparison of the relative strength of the correlation between urbanization and hydrologic metrics with B-IBI scores, it would also be instructive to look at the slopes and prediction confidence intervals among the metrics that best correlate with B-IBI and include a comparison with the first hydrologic PC1 (Clausen and Biggs, 1997). In Figure 6 we show the least-squares fit regression line and the 95% prediction confidence intervals for %TIA, Low Pulse Count, Low Pulse Duration, High Pulse Count, High Pulse Duration, High Pulse Range, Flow Reversals, *T*_Qmean_, R-B Index, and PC1. The regressions with the highest slope and smallest prediction confidence intervals are High Pulse Count, High Pulse Range, and PC1.

### Initial Application of Selected Indicators – Miller Creek Basin Plan

Based on the Mann-Whitney *U*-test, we determined that the Miller Creek HSPF model could reliably predict six of the eight hydrologic metrics that were also significantly correlated with B-IBI scores – the two Low Pulse metrics, the three High Pulse metrics, and *T*_Qmean_. The model did not reliably predict Flow Reversals or the R-B Index based on the Mann-Whitney test.

Comparison of the six reliably predicted hydrologic indicators calculated from the basin planning model results for fully forested, current, plan goal, and the preferred planning alternative indicated that the plan goal of 75/15/10 flow matching could be achieved with the preferred alternative (Executive Committee, 2006). However, the difference between the plan goal and fully forested conditions for *T*_Qmean_ and High Pulse Duration was relatively small (10-25% absolute difference), while the difference between the plan goal and fully forested conditions for the Low Pulse Count and Duration and High Pulse Count and Range metrics was still substantial – 70-270% difference (Table 7).

**TABLE 7:** Comparison of Low and High Pulse Metrics and *T*_Qmean_ Under Modeled Fully Forested Condition, Current (1995) Conditions, and Two Management Scenarios Based on Continuous Hydrologic Modeling Conducted for the Miller Creek Basin Plan.

Metric	Units	Fully Forested	Current Conditions	Plan Goal	Preferred Alternative
Low Pulse Count	# per year	3	13 (333)	11 (267)	10 (233)
Low Pulse Duration	Days	44	11 (−50)	13 (−70)	13 (−70)
Low Pulse Range	Days	136	193 (42)	204 (50)	193 (42)
High Pulse Count	# per year	7	25 (72)	17 (143)	16 (129)
High Pulse Duration	Days	4	3 (−25)	3 (−25)	3 (−25)
High Pulse Range	Days	99	317 (220)	266 (169)	256 (168)
*T*_Qmean_	Fraction of year	0.34	0.26 (−24)	0.31 (−9)	0.31 (−9)

Notes: Results presented are averages of model results for the period 1950 to 2002 and the percent difference with fully forested conditions is shown in parentheses. The Plan Goal (75/15/10) reflects a practical management goal in this basin, which assumes a target of 75% forest, 15% grass, and 10% impervious cover (Level 2 or 75/15/10) throughout the basin for all existing development and existing regional control facilities in place. The Preferred Alternative includes Level 2 (75/15/10) flow management requirements for new development and additional storage at regional detention facilities.

## Discussion

Our results suggest that the hydrology of urbanizing basins in the Puget Lowlands has a significant influence on the biotic integrity of streams. Eight of the fifteen metrics we evaluated were significantly correlated with B-IBI scores (Low Pulse Count, Low Pulse Duration, High Pulse Count, High Pulse Duration, High Pulse Range, Flow Reversals, *T*_Qmean_, and R-B Index) and all but two (Low Pulse Count and *T*_Qmean_) were significantly correlated with measures of urbanization.

High Pulse Count and High Pulse Range are measures of frequency and the period of time each year that high pulse events occur. We re-evaluated trends in High Pulse Count separately for wet (October to March) and dry (April to September) periods in the two urbanizing and one rural basin and found significant trends in wet period pulse counts in one urbanizing basin (Juanita, tau = 0.410; *p*=0.006) and in both urbanizing basins for dry period pulse counts (Juanita, tau = 0.301; *p*=0.035; Kelsey, tau = 0.477; *p*<0.00001) and no significant trends in wet or dry periods (*p*>0.05) in the largely rural and forested Issaquah basin. It appears that High Pulse Count increases as a result of increasing numbers of high pulses during winter and the occurrence of high pulses in summer as a basin becomes increasingly urbanized more summer high pulses occur than would have occurred historically.

Although the PCA indicated a great deal of redundancy among the eight potential hydrologic indicators, the ability of High Pulse Count and High Pulse Range to satisfy the four management characteristics, combined with the results of the comparison of the predictive capability of each hydrologic metric, suggests that High Pulse Count and High Pulse Range are the individual hydrologic metrics with the greatest potential for biological relevance. We should note that even though these metrics displayed the smallest B-IBI prediction confidence intervals (along with PC1), these prediction intervals span over half the range in B-IBI scores (see Figure 6) suggesting that at best these metrics could discriminate between the worst and best B-IBI locations.

We applied a fairly conservative test for statistical significance (Benjamini and Hochberg False Discovery Rate control) to minimize Type I and II errors in the identification of metrics related to urbanization and biotic integrity. The *p*-values for the correlations between %TIA and Low Pulse Count and *T*_Qmean_ were 0.035 and 0.036, respectively. A significant correlation between *T*_Qmean_ and %TIA in Puget Lowland streams has been noted previously (Booth *et al.*, 2004). It is likely that our statistical approach was overly conservative in this case and that the correlation between %TIA and *T*_Qmean_ is not spurious.

We were unable to eliminate the possibility that some basin characteristics (%Outwash, Basin Elevation, and Precipitation) potentially confound the relationships identified (or potentially explain some of the residual variation) between hydrologic metrics and urbanization and hydrologic metrics and B-IBI scores. These three potential confounding variables were also found to be correlated with measures of urbanization and B-IBI scores (%Outwash, Basin Elevation, and Precipitation). The correlation between elevation and B-IBI scores has been noted previously and attributed to the concentration of forest clearing and development in lowlands and less disturbance at higher elevations, resulting in a statistical, but unlikely causal, relationship between elevation and B-IBI (Fore *et al.*, 1996; Morley and Karr, 2002). We suggest that the relationship with %Outwash and Precipitation is caused by the same covariation of development with elevation and concentration of development along streams, rivers, and lakes where outwash deposits are typically found.

Local Channel Slope may be a confounding factor for High Pulse Duration, and Basin Area appears to be a confounding factor for Flow Reversals, *T*_Qmean_, and R-B Index. However, %Till does not appear to be a confounding factor as it was not significantly correlated with the eight hydrologic metrics or with measures of urbanization or B-IBI scores. The potentially confounding relationships between Basin Area and *T*_Qmean_ (Konrad and Booth, 2002) and R-B Index (Baker *et al.*, 2004) have been noted previously, and consideration should be given to controlling for these effects if these metrics are used for management or further research into flow-ecology relationships.

Unfortunately, we could not test the potential confounding effect of %Bedrock because of its non-normal distribution in our dataset. Only seven of the 16 basins contained measurable amounts of bedrock ranging from 4% to 31%. The highest sub-basin (Issaquah near Hobart) with 20% bedrock had the lowest %TIA, but a “fair” B-IBI score of 32, more consistent with the response to the potential hydrologic indicators than to %TIA (Figure 6), providing some circumstantial evidence that bedrock and/or elevation/precipitation driven hydrologic differences were a significant factor controlling the biological integrity of this stream. The lower elevation sub-basin with the highest amount of bedrock (May Creek) was moderately urbanized (%TIA = 24.5) and had a “poor” B-IBI score of 24 that appeared fairly consistent with %TIA and the potential hydrologic indicators (Figure 6), suggesting that the hydrologic differences in Issaquah near Hobart are due more to elevation/precipitation than to bedrock.

The PCA results indicate that there is a great deal of redundancy among these eight metrics with respect to their response to urbanization. We suggest that the eight metrics found to be correlated with B-IBI scores are all surrogate measures of the increase in the frequency of occurrence of high flow pulses in winter and summer and associated low flow pulses during summer. These high and low flow summer pulses did not typically occur under historically forested conditions. Benthic invertebrates that are best able to withstand these flow disturbances (i.e., small, mobile, short-lived species that have multiple reproductive cycles throughout the year – multivoltine species), would become more abundant than larger, less mobile species (with only one or two annual reproductive cycles – univoltine or semivoltine species). Consistent with this hypothesized disturbance mechanism, mayflies of the genus *Baetis* (many of which are small and multivoltine) occur with greater relative abundance in our more urbanized streams (Cassin *et al.*, 2005). Dominance of samples by a few mayfly (Ephemeroptera) taxa that are not clinger or predator taxa; a lack of stoneflies, caddis flies, and generally intolerant long-lived species; and a high percentage of tolerant taxa – typically taxa in the Plenariidae (flatworms), Hirudinea (leeches) or Simuliidae (black flies) – results in lower B-IBI scores.

Six of the eight hydrologic metrics correlated with B-IBI also consistently identified trends over time in two urbanizing basins (Juanita and Kelsey) with long-term data records – Low Pulse Count, Low Pulse Duration, High Pulse Range, High Pulse Count, *T*_Qmean_, and R-B Index. Consistent with the observations of Baker *et al.* (2004), R-B Index appeared to the most sensitive trend detection metric (highest tau values) due to its low inter-annual variability and clear response to the hydrologic effects of urbanization. Baker *et al.* (2004) found that the inter-annual variability of the R-B Index was much lower than most IHA metrics and had much greater power to detect trends in flow flashiness based on 100 randomly selected stream gauges from six Midwestern states. Detected trends in *T*_Qmean_ in Juanita and Kelsey Creeks (and lack of trend in Issaquah Creek) confirm the trends in *T*_Qmean_ previously identified by Konrad and Booth (2002) in the same creeks. Although R-B Index appears to be the most sensitive metric for detecting trends related to urbanization based on our limited evaluation, it is one of the weakest “predictors” of B-IBI scores – on par with the urbanization measures %TIA and %Urban. We should also emphasize that flow monitoring for the purpose of trend detection will likely only identify impacts long after they have occurred or will be confounded by the large inter-annual climate variability and the time window available for trend analysis (Konrad and Booth, 2002).

We noted a consistent significant inverse relationship between several of our hydrologic metrics and urbanization measures and non-urban forest cover. The inverse relationship between urbanization measures and non-urban forest cover has been recognized previously with respect to phosphorus concentrations in King County streams (Brett *et al.*, 2005; Alberti *et al.*, 2007). The strong inverse relationship between urbanization and forest cover has been attributed to the predominant mode of land transformation from forest to suburban and urban development in King County (Brett *et al.*, 2005). The correlation between %TIA and %Urban with %Forest in our basins was −0.998 (*p*<0.0001) and −0.987 (*p*<0.0001), respectively.

*T*_Qmean_ is the only hydrologic metric we have selected that has already been shown to have a statistically significant linear relationship with B-IBI scores from the Puget Lowland (Morley and Karr, 2002; Booth *et al.*, 2004). The most recent research has consistently pointed to the importance of the connectedness of impervious surfaces to streams as urban areas expand (Hatt *et al.*, 2004; Walsh, 2004). This has resulted in a shift from the most general measures (or surrogates) of urbanization such as impervious surface cover (%TIA; Booth *et al.*, 2004; Alberti *et al.*, 2007; Matzen and Berge, 2008) or percent of urbanized area (%Urban; Morley and Karr, 2002) to road crossings/road density (Konrad *et al.*, 2005; Alberti *et al.*, 2007), hydrologic metrics (Booth *et al.*, 2004), and number of stormwater connections to the stream (Walsh, 2004). Here we have provided further evidence for the suggestion of Booth *et al.* (2004) that hydrologic metrics may be a more direct measure of the effects of urbanization on stream biota – specifically benthic macroinvertebrates.

Although our study does not provide empirical evidence for mechanistic links (i.e., cause and effect) between summer flow pulses and B-IBI scores, our findings support the hypothesis that species composition of macroinvertebrates in Puget Lowland streams now favors species tolerant of summer flow pulses that did not occur historically. We believe our study results provide a starting point for further research that could test our hypothesis and further refine our understanding of the mechanisms involved.

### Case Study Application

**FIGURE 7:**
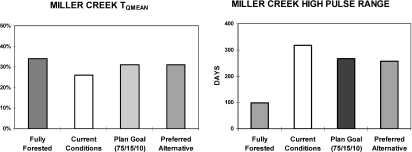
Comparison of *T*_Qmean_ and High Pulse Range Under Modeled Fully Forested Condition, Current (1995) Conditions, and Two Management Scenarios – Plan Goal and the Preferred Alternative. The Plan Goal reflects a practical management goal in this basin, which assumes a target of 75% forest, 15% grass, and 10% impervious surface cover (75/15/10) throughout the basin with existing regional control facilities in place. The Preferred Alternative includes 75/15/10 flow management requirements for new development and additional storage at regional detention facilities.

Modeled Miller Creek Basin planning alternatives for *T*_Qmean_ and High Pulse Range, illustrate the ability of the preferred alternative to meet the plan goal and the disparity between the plan goal and fully forested conditions for these metrics (Figure 7). The difference between the preferred management alternative and fully forested conditions for *T*_Qmean_ was relatively small, while the difference between the preferred management alternative and fully forested conditions for the Low and High Pulse metrics was still rather large, suggesting that the biological benefit (if the pulse metrics are truly a predictor of biological condition and the effect of development on stream biota can be reversed) of the preferred management alternative may be less than what is suggested based on consideration of *T*_Qmean_ alone.

Additional model calibration would be required to more reliably predict Flow Reversals and R-B Index so they could also be compared. We suspect that pulses are easier to predict as it is necessary only to capture the exceedance of the pulse thresholds rather than match a particular flow magnitude or duration.

We do not mean to imply that restoration in such a highly urbanized basin should strive for complete hydrologic restoration (see Booth *et al.*, 2004), but we want to draw attention to the fact that regulating runoff from new development and improvements in performance of a large regional detention facility can influence *T*_Qmean_, but has much less influence on the Low and High Pulse metrics.

Historically, mitigation of the impacts of development on stream hydrology has focused narrowly on structural and site-specific end-of-pipe remedies (Booth *et al.*, 2002). Although this approach has reduced peak flows and mitigated flooding and erosion problems (Booth *et al.*, 2002), there is little evidence that this approach has effectively protected biological resources downstream of these measures (Maxted and Shaver, 1997; Horner *et al.*, 1999, 2002; Maxted, 1999). Furthermore, mitigation requirements have applied only to new development and exceptions are granted for developments below a certain threshold, which on a parcel scale may result in insignificant impacts, but on a cumulative basin-scale may be significant (Booth and Jackson, 1997). If development is socially desired, focus should be on maintaining natural flow patterns (including summer months) at the basin-scale and avoidance of direct discharge to streams (Walsh *et al.*, 2005).

### Implications for Modeling

Given the difficulty of separating urbanization effects from other factors (climate, basin area, soils, geology, elevation, precipitation, etc.) that weaken the ability to unequivocally identify hydrologic indicators for management purposes (and the lack of predisturbance B-IBI and flow data), a hydrologic modeling approach in addition to the gauge-data approach used in our study might prove to be very useful (Richter *et al.*, 1998; Poff *et al.*, 2006; Sanborn and Bledsoe, 2006). For example, it might be helpful to use models to extrapolate flow metrics to B-IBI sampling locations that are not located near existing flow gauges (e.g., Cassin *et al.*, 2005). There are far more historical B-IBI sampling locations in the Puget Lowland than we have used in this study. For example, Alberti *et al.* (2007) evaluated relationships between B-IBI and a variety of urbanization patterns using a dataset consisting of 36 separate B-IBI locations in 14 distinct Puget Lowland stream systems, but noted the general lack of gauge records for their sampling sites. A broader selection of sites would allow further testing and identification of flow-ecology relationships. This approach would also provide datasets suitable for construction of statistical models that might control for confounding variables or include additional explanatory variables as no one cause or variable will likely explain all of the variation in B-IBI scores (Alberti *et al.*, 2007).

In King County, long-term hydrologic modeling of current, future, and fully forested conditions (as described above) has been the foundation of basin planning for the past 20 years. This modeling approach provides the data (albeit synthetic) needed to calculate our annual hydrologic metrics and compare the results obtained from models of fully forested and current conditions and any modeled management scenario (see the Miller Creek Basin Plan example above). The *range of variability* approach described by Richter *et al.* (1998) using modeled predevelopment (fully forested) and postdevelopment (current or management scenario) conditions to quantify the *degree of hydrologic alteration* (Shiau and Wu, 2004) within a particular basin might be another way to use models to control for the effects of variation in climate and basin-specific characteristics as part of a basin-scale regional water resources assessment program.

### Implications for Other Regions

Although the hydrologic response to urbanization in any particular region depends on a variety of factors that include development types and patterns, hydroclimate, geology, physiography, vegetation, and catchment size (Poff *et al.*, 2006), we would expect to see a biological response in regions where urbanization affects normally stable summer base-flows. We also suspect that our conceptual approach to linking hydrologic alteration to biological impairment in urbanizing streams could be adapted for use in other regions with different flow regimes and biological communities to help filter the large number of flow metrics down to those that are most likely to be biologically relevant.

## Conclusions

Our initial criteria for selecting hydrologic indicators identified eight hydrologic metrics that are significantly correlated with a measure of stream biological condition (Pacific Northwest B-IBI) and six of these hydrologic metrics were significantly correlated with measures of urbanization. However, only six of the eight metrics consistently demonstrated trends in urbanizing basins (Low Pulse Count, Low Pulse Duration, High Pulse Count, High Pulse Range, *T*_Qmean_, and R-B Index). Three of eight indices (Flow Reversals, *T*_Qmean_, and R-B Index) were compromised by their correlation with basin area and one was compromised by correlation with Local Channel Slope (High Pulse Duration). Only two of the hydrologic metrics tested, High Pulse Count and High Pulse Range met all four criteria – they were most highly correlated with B-IBI and measures of urbanization (%TIA and %Urban), reliably detected trends and were not confounded by basin area. The increase in these high pulse metrics with respect to urbanization is the result of an increase in winter high pulses and the occurrence of high pulse events during summer (increasing the frequency and range of high pulses), when practically none would have occurred prior to development.

If future research continues to support this hypothesis, it would have significant implications for stormwater management in Puget Lowland streams. Stream biota might be better protected by minimizing the amount of new land developed and minimizing the number of direct connections and the amount of runoff from impervious surfaces. Low Impact Development approaches might be an attractive approach to minimizing summer runoff as the volume of water delivered during this period in the Puget Lowland is much smaller relative to winter runoff volumes (Konrad and Burges, 2001; Holman-Dodds *et al.*, 2003; Hood *et al.*, 2007). Low Impact Development approaches, especially those that include infiltration of runoff, have the added benefit of potentially reducing the toxic effects of contaminated runoff. Social, economic, and political measures to preserve existing forest cover should not be overlooked as preserving the historical landscape (i.e., forest and soils) provides the historical flow regime that in turn provides the highest degree of certainty that native biological communities will be adequately protected.

## SUPPORTING INFORMATION

Table S1. Summary Statistics for the 16 Study Basins, Including the 15 Hydrologic Metrics and B-IBI Scores.

Please note: Neither AWRA nor Wiley-Blackwell is responsible for the content or functionality of any supporting materials supplied by the authors. Any queries (other than missing material) should be directed to the corresponding author for the article.
